# Fabrication of Microfibrous and Nano-/Microfibrous Scaffolds: Melt and Hybrid Electrospinning and Surface Modification of Poly(L-lactic acid) with Plasticizer

**DOI:** 10.1155/2013/309048

**Published:** 2013-12-05

**Authors:** Young Il Yoon, Ko Eun Park, Seung Jin Lee, Won Ho Park

**Affiliations:** ^1^Department of Advance Organic Materials and Textile System Engineering, Chungnam National University, Daejeon 305-764, Republic of Korea; ^2^College of Pharmacy, Ewha Womans University, Seoul 120-750, Republic of Korea

## Abstract

Biodegradable poly(L-lactic acid) (PLA) fibrous scaffolds were prepared by electrospinning from a PLA melt containing poly(ethylene glycol) (PEG) as a plasticizer to obtain thinner fibers. The effects of PEG on the melt electrospinning of PLA were examined in terms of the melt viscosity and fiber diameter. Among the parameters, the content of PEG had a more significant effect on the average fiber diameter and its distribution than those of the spinning temperature. Furthermore, nano-/microfibrous silk fibroin (SF)/PLA and PLA/PLA composite scaffolds were fabricated by hybrid electrospinning, which involved a combination of solution electrospinning and melt electrospinning. The SF/PLA (20/80) scaffolds consisted of a randomly oriented structure of PLA microfibers (average fiber diameter = 8.9 *µ*m) and SF nanofibers (average fiber diameter = 820 nm). The PLA nano-/microfiber (20/80) scaffolds were found to have similar pore parameters to the PLA microfiber scaffolds. The PLA scaffolds were treated with plasma in the presence of either oxygen or ammonia gas to modify the surface of the fibers. This approach of controlling the surface properties and diameter of fibers could be useful in the design and tailoring of novel scaffolds for tissue engineering.

## 1. Introduction

Electrospinning from melt is an attractive solvent-free manufacturing process for tissue engineering scaffolds [1]. Melt electrospinning has inherent advantages, such as cleaner processing with environmental safety and higher productivity (with lower production cost) due to the absence of solvent. In addition, melt electrospinning is particularly attractive for its applicability to commodity polymers, such as polypropylene and polyethylene, which are only soluble in limited solvents and require high temperature for dissolution [2–7]. However, a polymer melt has higher viscosity than the solution state, which usually generates thicker micron-sized fibers than those of solution electrospinning. In addition, the tendency of the polymer to solidify as it flows out of a nozzle hinders the stretching of the polymer jet, thus resulting in microfibers [8].

In order to fabricate thinner fibers via melt electrospinning, the viscosity of a polymer melt should be reduced by adjusting the processing parameters or polymer parameters. Important parameters reported for melt electrospinning are the molecular weight, tacticity, melting point of the polymer, electric field strength, distance from the nozzle, mass flow rate, and process temperatures (electrospinning temperature, heating chamber temperature) [9–13]. Detta et al. [14] melt electrospun a novel blend of a high molecular weight polymer, poly(*ε*-caprolactone), with a low molecular weight polymer, poly(ethylene glycol)-*block*-poly(*ε*-caprolactone). It was noted that the diameter of the fiber obtained was in the micron range and was directly related to the mass flow rate. Dalton et al. [9] employed a viscosity-reducing additive to reduce the diameter of fiber electrospun from the polymer melt. They found that the additive reduces the average chain length and thus reduces the viscosity of the polymer solution and, with it, the fiber diameter.

Nanofibrous structures produced by electrospinning (with nanosized fiber diameter, appropriate pore size, and high surface area) are very useful in the design of scaffolds for tissue engineering via higher cell absorption on a material. Melt electrospinning can be a better technology for fibrous scaffolds, because it avoids toxic solvents. Several polymers to date have been melt electrospun into microfibrous scaffolds for tissue engineering applications [15–17]. Poly(lactic acid) (PLA) is one of the most widely used synthetic polymers in biomedical applications. PLA has been widely used in the areas of surgical suture, implant materials, drug carriers, and scaffolds for tissue engineering [18, 19]. However, PLA has poor hydrophilicity, and no natural cell recognition sites exist on its surface [20].

This study examined the melt- and hybrid electrospinning of PLA, which is a promising biomaterial for scaffolds with good biocompatibility and biodegradability. To this end, a plasticizer additive for the PLA melt was used to reduce the viscosity of the polymer melt and decrease the fiber diameter. Also, the effects of the processing parameters, such as spinning (syringe) temperature (*T*_*s*_), mass flow rate (*Q*), and heating chamber temperature (atmospheric temperature), were investigated. In addition, the melt- and hybrid electrospun PLA fibers were treated with oxygen or ammonia plasma in order to improve their surface hydrophilicity. Changes in the surface characteristics, including the hydrophilicity and chemical composition, were investigated using contact angle measurement, moisture content, and X-ray photoelectron spectroscopy.

## 2. Materials and Methods

### 2.1. Materials

PLA (*M*_*v*_ = 70,000) with *L* content >95% was supplied by Huvis Co., Korea. PEG (*M*_*n*_ = 2,000) was purchased from Yakuri Pure Chemicals Co., Japan, and used as received. Raw 12-denier silk fibers (*B. mori*) were supplied by Daejeon Sangsa Co., Korea, and silk fibroin (SF) was regenerated according to the methods in a previous study [21]. Dimethyl formamide (DMF), chloroform, and 1,1,1,3,3,3-hexafluoro-2-propanol (HFIP) were purchased from Sigma-Aldrich Co., USA, and used without further purification.

### 2.2. Melt- and Hybrid Electrospinning

**Figure 1:**
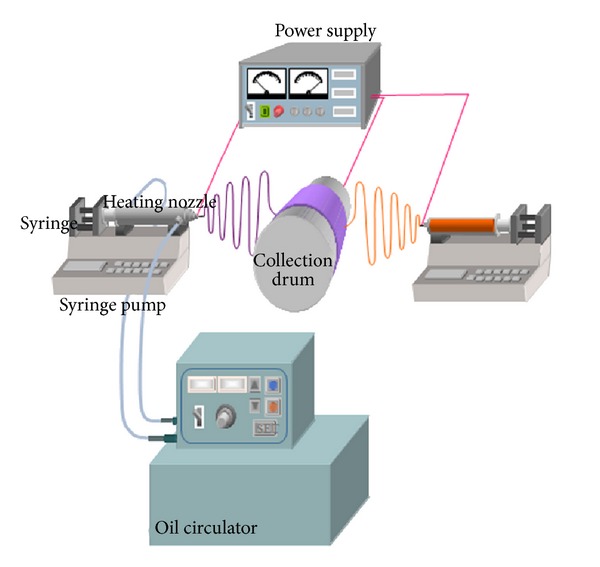
Schematic diagram of the hybrid electrospinning apparatus.

Figure 1 shows a schematic diagram of the hybrid electrospinning apparatus (Nano NC, Korea). The spinneret for melt electrospinning consisted of a stainless steel tube capable of oil circulation and a glass syringe placed inside the stainless steel tube. The temperature of the glass syringe was controlled using an oil circulator with a temperature controller. In the melt electrospinning process, PLA microfibers were prepared by electrospinning a PLA melt at temperatures ranging from 185 to 225°C and were collected on a target drum at a distance of 8 cm from the syringe tip (21 G, 0.495 mm). A voltage of 21 kV was applied to the collecting target using a high-voltage power supply (Chungpa EMT, CPS-40K03), and the mass flow rate of the melt was varied from 0.65 to 5.40 mL/h using a syringe pump (KD Scientific, Model 100). The atmosphere temperature in the heating chamber was controlled in the range from 25 to 65°C.

In the solution electrospinning compartment, the PLA and SF nanofibers were prepared by electrospinning a PLA solution (14 wt%) in chloroform/DMF (5/1, v/v) and an SF solution (7 wt%) in HFIP, respectively. The nanofibers were collected on a target drum placed 5 cm from the syringe tip (21 G). A voltage of 8 kV was applied to the collecting target by a high-voltage power supply, and the flow rate of the solution was 4 mL/h.

For the PLA/PLA or SF/PLA nano-/microfiber composite scaffolds, a PLA solution (14 wt%) and SF solution (7 wt%) and PLA melt were electrospun simultaneously in opposite directions facing the rotating target. The compositions (10/90, 20/80, 30/70, w/w) of the nano-/microfiber composite scaffold were controlled by adjusting the mass flow rate of the PLA melt from 0.65 to 5.40 mL/h at a fixed flow rate of the solution (4 mL/h). The melt- and hybrid electrospinning conditions for PLA microfibrous and nano-/microfibrous scaffolds are summarized in Table 1.

### 2.3. Plasma Treatment

Plasma treatment is known to be an environmentally friendly process to improve the surface hydrophilicity of polymer scaffolds. The O_2_ or NH_3_ plasma treatments were carried out with the melt electrospun PLA or hybrid electrospun PLA/PLA fibers in a chamber (36 cm × 25 cm × 15 cm) connected to a two-stage rotary pump via a liquid nitrogen cold trap with a base pressure of 4 × 10^−3^ mbar. An L-S matching unit was used to minimize the standing wave ratio (SWR) of the power transmitted from the 13.56 MHz radio frequency generator. Prior to each plasma treatment, the chamber was cleaned using 50 W air plasma for 30 min. A piece of the PLA fibers was then placed at the center of the chamber, followed by evacuation to the base pressure. O_2_ or NH_3_ gases were admitted into the system through a needle valve at a pressure of 0.2 mbar, and the electrical discharge was initiated. To detect the effect of plasma gas on the PLA fiber, the plasma treatment was carried out under the conditions for 0–300 sec with O_2_ gas and for 0–60 sec with NH_3_ gas, respectively. Upon the completion of surface modification, the gas feed was turned off and the chamber was vented to the atmosphere. All plasma treatments were carried out at room temperature (22 ± 1°C).

### 2.4. Moisture Content

The moisture content of the melt electrospun PGA microfibers and hybrid electrospun composite fibers (200 mg) was determined by immersing the fibers in distilled water for 1 h at room temperature [22]. The hydrated samples were then taken out and immediately weighed after removing the surface water with filter paper. The water content (WC, %) was calculated as follows: WC(%) = (*W* − *W*_0_)/*W*_0_ × 100, where *W*_0_ and *W* denote the weight of the sample before and after immersion in water for 1 h, respectively.

### 2.5. Measurements and Characterization

The melt viscosity was measured using a rheometer (ARES, Rheumatics, USA) with a shear rate of 10 rad/sec from 150 to 210°C. The morphology of electrospun PLA fibers was observed by field emission scanning electron microscopy (FE-SEM, JSM-7000F, JEOL, Japan). Prior to the observations, the samples were coated with platinum by ion sputtering for a few seconds. The average fiber diameter and diameter distribution were obtained by analyzing the SEM images with a custom-code image analysis program (Scope Eye II, Korea). The porosity and pore parameters in the interfiber region of PLA fibers were determined using a mercury intrusion technique with an AutoPore III mercury porosimeter (Micromeritics Instrument, Norcross, GA, USA). Differential scanning calorimetry (DSC) was conducted using a TA instruments 2920 (duPont Co.). Samples were heated from 20°C to 200°C at a rate of 10°C/min (first heating) and held at the final temperature for 1 min to eliminate the thermal history applied to the samples. After cooling to −100°C, they were then reheated to 200°C at a rate of 10°C/min (second heating). The glass transition temperature (*T*_*g*_), melting temperature (*T*_*m*_), and cold crystallization temperature (*T*_*c*_) were obtained from the second run. The mechanical properties of the electrospun PLA scaffolds were measured using an Instron tensile tester (Instron 8511, Canton, MA, USA) at a crosshead speed of 5 mm/min. The samples were prepared using the D-638-5 ASTM method and tested at 25°C and 50% humidity (*n* = 10). The contact angle of water droplets on the samples was measured using a DSA100 Drop Shape Analyzer System (KRÜSS, Germany). Deionized water was used, and ten independent measurements were averaged. The surface chemical composition of PLA fibers was investigated before and after plasma treatment using X-ray photoelectron spectroscopy (XPS). XPS spectra of the plasma-treated samples were acquired on an ESCALAB 250 XPS spectrometer (VG Scientific, USA).

## 3. Results and Discussion

### 3.1. Effect of Plasticizer on the Thermal Properties of PLA

**Figure 2:**
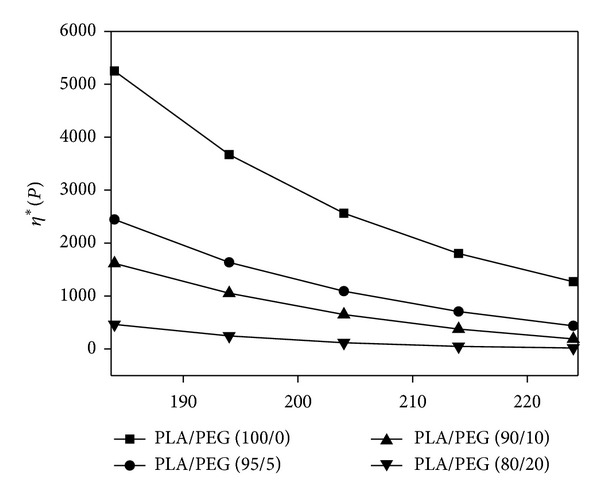
Melt viscosity of PLA containing plasticizer (PEG) as a function of temperature.

**Figure 3:**
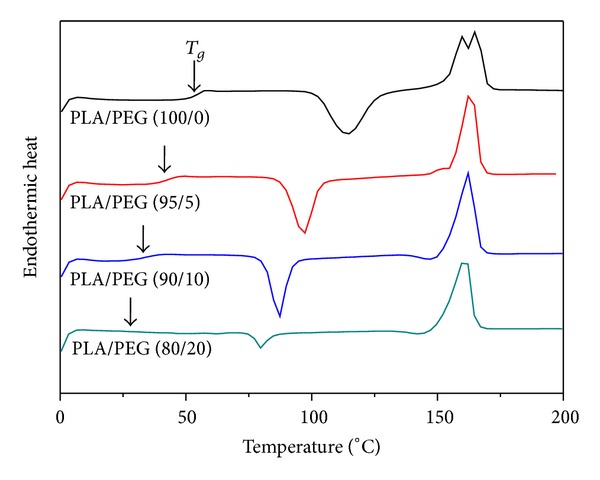
DSC thermograms obtained from a second heating for PLA/PEG blends.

The melt electrospinning process is strongly affected by the viscosity of the polymer melt, which is strongly dependent on the temperature for thermoplastic polymers. Therefore, it is important to examine the melt viscosity of a polymer in the temperature range of possible processing.  Figure 2 shows the change in melt viscosity of PLA with temperature and PEG plasticizer content. The melt viscosity of neat PLA was 5,300 Poise at 185°C and gradually decreased to 500 Poise with increasing PEG content up to 20 wt%. The melt viscosities of PLA samples decreased gradually with increasing temperature from 185 to 225°C. The difference in melt viscosity between 185°C and 225°C was approximately 3 times, compared with the neat PLA sample, and the differences were narrower with increasing PEG content up to 20 wt%. To investigate the efficiency of the PEG on PLA, DSC was carried out using the PLA blends with different PEG content up to 20 wt%.  Figure 3 shows the DSC thermograms obtained from a second heating for PLA/PEG blends. A gradual depression of the glass transition temperature (*T*_*g*_) from 50 to 25°C was observed with increasing PEG content. Effective plasticization induces the depression of the glass transition temperature in the polymer/plasticizer system. Therefore, PEG was expected to be compatible with PLA. The cold crystallization temperature (*T*_*c*_) of PLA was lower than that of neat PLA, while the melting temperatures (*T*_*m*_) were nearly unchanged.

### 3.2. Effect of Plasticizer on the Diameter of Melt Electrospun PLA Fibers

**Figure 4:**
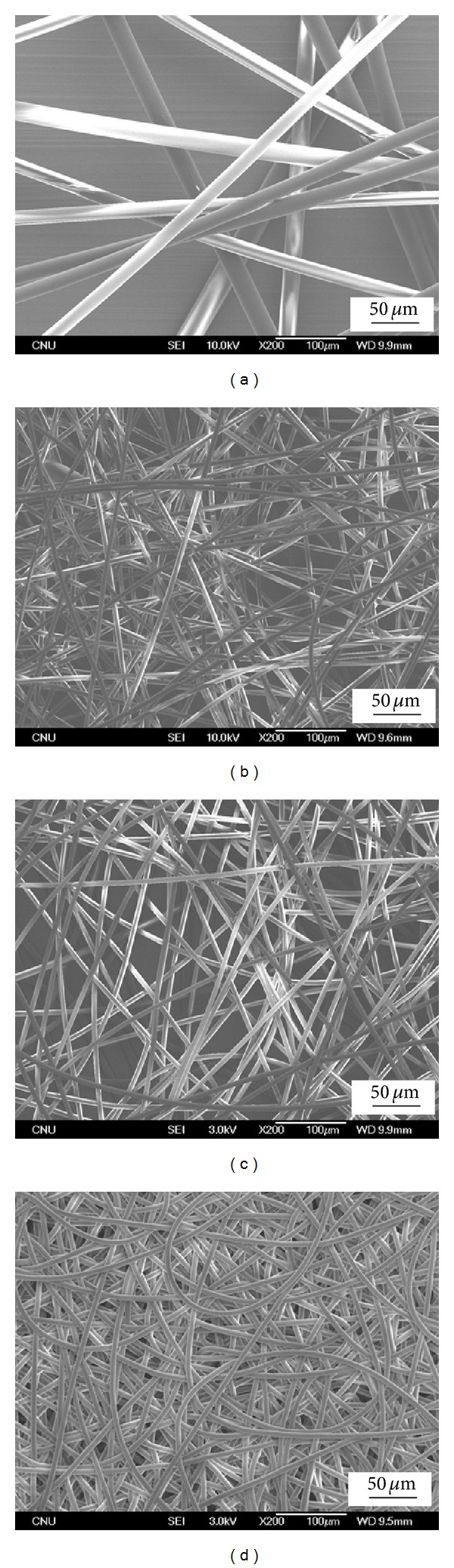
SEM images of the melt electrospun PLA fibers with different PEG contents: (a) PLA/PEG (100/0), (b) PLA/PEG (95/5), (c) PLA/PEG (90/10), and (d) PLA/PEG (80/20).

Figure 4 shows representative SEM images of the melt electrospun PLA fibers with different PEG contents at syringe temperatures of 215°C. The average diameter and deviation of electrospun PLA fibers decreased significantly from 24.7 ± 3.8 to 8.97 ± 1.63 by adding 5% of PEG (Figures 4(a)-4(b)), but a slight decrease was observed when further increasing PEG contents from 5% to 20% (Figures 4(b)–4(d)). This seems to be associated with the change in viscosity of PLA melt containing PEG plasticizer, as shown in Figure 2. Therefore, it was found that the plasticizer content is a critical parameter affecting the fiber diameter and morphology.

### 3.3. Effect of Atmospheric Temperature on the Diameter of Melt Electrospun PLA Fibers

**Figure 5:**
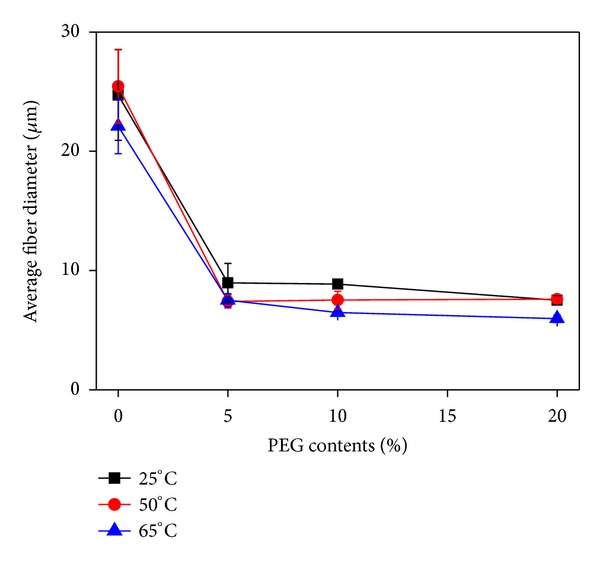
Change in the average diameter of PLA fibers with different PEG contents as a function of atmosphere temperature.

The atmospheric temperature determined from the glass transition region of neat PLA was varied from 25 to 65°C in order to investigate its effect on the fiber diameter of melt electrospun PLA fibers.  Figure 5 shows the change in the average diameter of PLA fibers with different PEG contents as a function of atmospheric temperature. The average diameter of PLA fibers and its standard deviation were decreased with increasing PEG content. Particularly, the addition of 5 wt% PEG to PLA induced a dramatic decrease in the average fiber diameter of PLA fibers. However, the effect of atmospheric temperature ranging from 25 to 65°C on the average diameter of PLA fibers was not significant.

### 3.4. Effect of Mass Flow Rate on the Diameter of Melt Electrospun PLA Fibers

**Figure 6:**
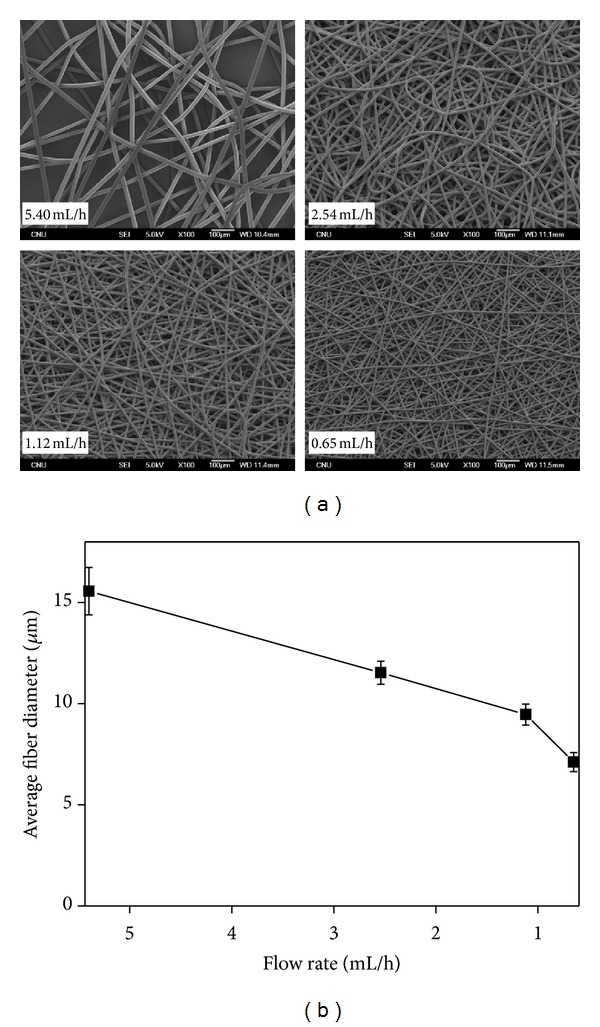
(a) SEM images and (b) change in the fiber diameter of the melt electrospun PLA fibers containing 10 wt% PEG at different mass flow rates.

In a previous study [23], the effects of processing parameters on the average diameter and morphology of melt electrospun PLGA fibers were examined. Among the processing parameters, the mass flow rate had the most influence on the fiber diameter of PLGA.  Figure 6(a) shows representative SEM images of the melt electrospun PLA fibers containing 10 wt% PEG at different mass flow rates of 5.40, 2.54, 1.12, and 0.65 mL/h. The average diameter (and standard deviation) of the PLA fibers decreased significantly by about half with decreasing mass flow rate from 5.4 to 0.65 mL/h.  Figure 6(b) shows the change in the fiber diameter of PLA with different mass flow rates under fixed spinning conditions. Interestingly, the average fiber diameter and its deviation decreased significantly with decreasing mass flow rate from 5.40 to 0.65 mL/h. This significant decrease in the fiber diameter at the lower mass flow rate may have been due to the formation of a smaller Taylor cone due to the decreased volume supplied from the polymer melt. In contrast, the fiber diameter was not influenced significantly by the flow rate in solution electrospinning.

### 3.5. PLA Composite Fibers from Hybrid Electrospinning

**Figure 7:**
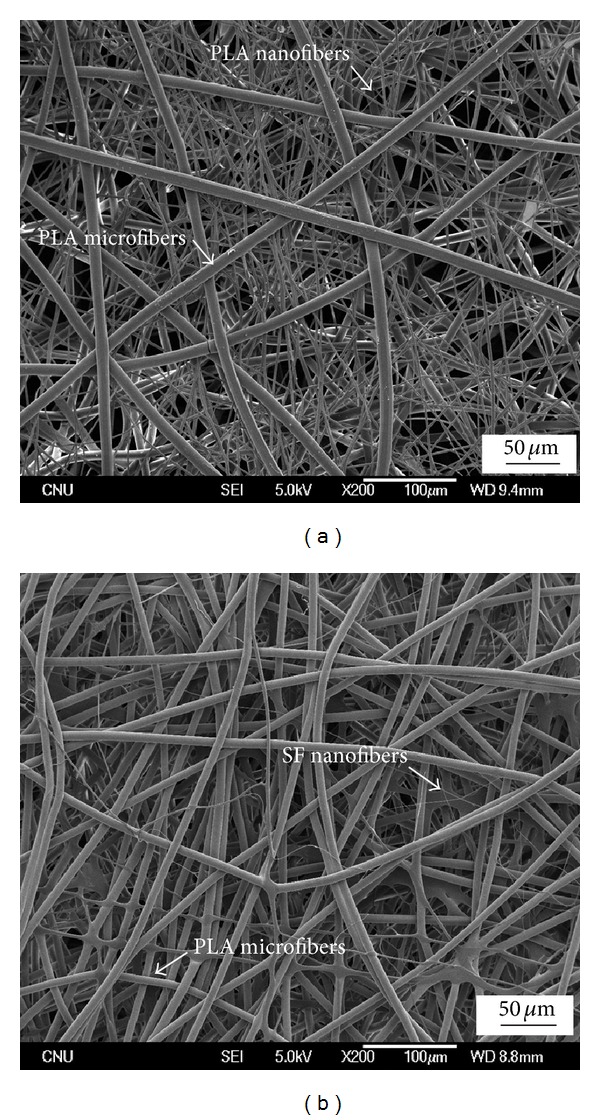
SEM images of (a) PLA/PLA (20/80) and (b) SF/PLA (20/80) nano-/microfiber composite scaffolds.

To fabricate a randomly mixed and interconnected network structure between the nanofibers and microfibers, a novel hybrid electrospinning process was designed, combining melt electrospinning with solution electrospinning [24]. This hybrid electrospinning could provide a randomly mixed nano-/microfibrous composite scaffold with higher pore diameters by simultaneous electrospinning.  Figure 7 shows SEM images of the PLA/PLA (20/80) and SF/PLA (20/80) nano-/microfiber composite scaffolds fabricated using PLA and SF solution and PLA melt under fixed electrospinning conditions. The PLA/PLA (20/80) and SF/PLA (20/80) nanofiber/microfiber composite scaffolds were fabricated using 14 wt% PLA and 7 wt% SF solution in the solution electrospinning compartment, respectively. In order to vary the composition of the nanofiber/microfiber composite scaffolds, the change in mass flow rate of the PLA melt was feasible and desirable because it is difficult to obtain a desired composition of nano-/microfiber scaffolds by changing the flow rate of the polymer solution. As shown in Figure 7, the PLA microfibers in nano-/microfiber composite scaffolds had a larger average diameter of 8.86 ± 0.25 *μ*m, whereas PLA and SF nanofibers had smaller average diameters of 1260 ± 230 nm and 820 ± 240 nm, respectively. The difference in average fiber diameters between the nanofibers and microfibers was approximately one order of magnitude.

The pore parameter (pore size, porosity) of the scaffolds is a crucial factor affecting the cell attachment, spreading, and migration.  Table 2 summarizes the pore parameters of the PLA microfibers and PLA nano-/microfiber (20/80) composite scaffolds determined by mercury porosimetry. The porosities of the PLA/PLA and SF/PLA nano-/microfiber (20/80) composite scaffolds were 91.6% and 95.0%, respectively, indicating high porosity. The total pore volumes of PLA/PLA and SF/PLA samples were 9.9 mL/g and 6.4 mL/g, respectively. In contrast, the porosity and pore volume of the PLA microfiber scaffolds were 94.2% and 13.3 mL/g, respectively. The average pore diameters (APD) in terms of the volumes (*V*) of the PLA/PLA nano-/microfiber (20/80) composite scaffold and PLA microfibrous scaffold were 34.5 *μ*m and 44.7 *μ*m, respectively. This decrease in pore diameter of the PLA/PLA composite scaffold might be due to the introduction of a small amount of nanofibers (20 wt%). However, a pore size of approximately 40 *μ*m is large enough to allow cells to freely migrate in many cases [25].

The mechanical properties of the PLA microfibers and PLA-based nano-/microfiber composite scaffold were assessed using a tensile test. The maximum load value of the PLA scaffolds was evaluated.  Table 2 shows the tensile strength and breaking elongation of the PLA microfibers and PLA composite scaffolds. The PLA/PLA (20/80) nano-/microfiber composite scaffold had higher tensile strength (26.8 gf/mm^2^) and modulus (2.7 gf/mm^2^) than those (1.5 gf/mm^2^ and 0.3 gf/mm^2^) of the PLA microfiber scaffolds. This may be explained by the nanofibrous structure entangled with microfibers. The nanofibers in the nano-/microfiber scaffolds can provide higher contacts or physical junctions with the microfibers or nanofibers and thus act as physical crosslinks.

### 3.6. Effect of Plasma Treatment on the Hydrophilicity of the Hybrid Electrospun PLA Fibers

Biodegradable aliphatic polyesters such as PLA and poly(*ε*-caprolactone) (PCL) are studied widely in scaffolds for tissue engineering. However, it is desirable to improve their surface properties because they have poor hydrophilicity and no functional sites for cell recognition. Plasma treatment provides an environmentally friendly process to improve the surface hydrophilicity of polymers without a serious loss of their bulk properties [26, 27].

**Figure 8:**
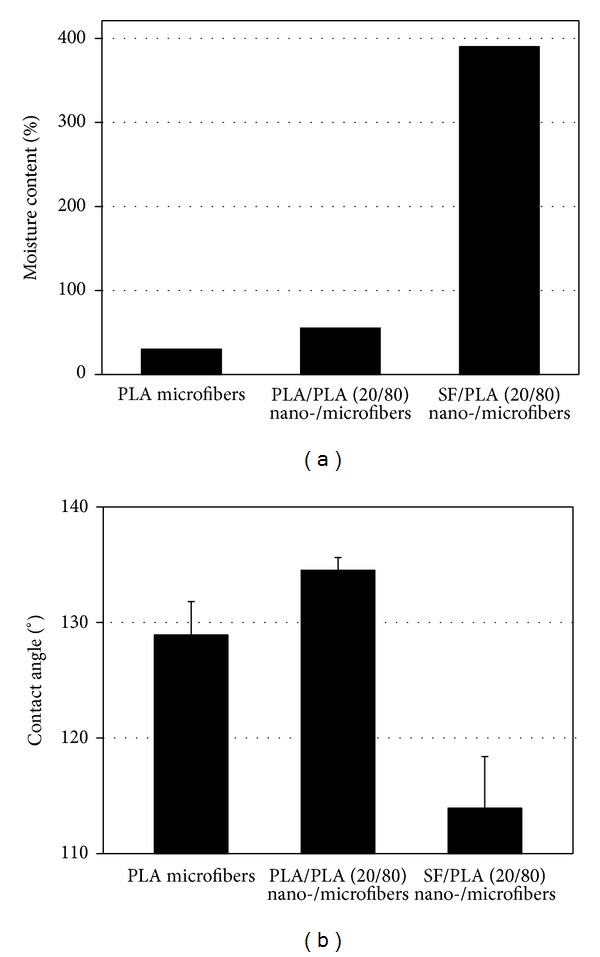
Moisture content (MC) and water contact angle (WCA) of PLA microfibers and PLA-based nano-/microfibers (20/80) composite scaffolds.

Figure 8 represents the moisture content (MC) and water contact angle (WCA) of PLA microfibers and PLA-based nano-/microfiber (20/80) composite scaffolds. Both PLA microfibers and PLA/PLA (20/80) composite fibers showed a low MC (30% for PLA, 55% for PLA/PLA) and a high WCA (129° for PLA, 135° for PLA/PLA), whereas SF/PLA (20/80) composite fibers showed a high MC (390%) together with a WCA of 114° because of the relatively hydrophilic SF. The higher MC in the PLA/PLA sample compared to the PLA microfibers may be associated with a higher surface area due to the combined nanofibrous structure. The WCA is strongly dependent on the surface roughness and surface hydrophilicity, and thus the higher WCA of PLA/PLA composite fibers may be affected by the surface roughness.

**Figure 9:**
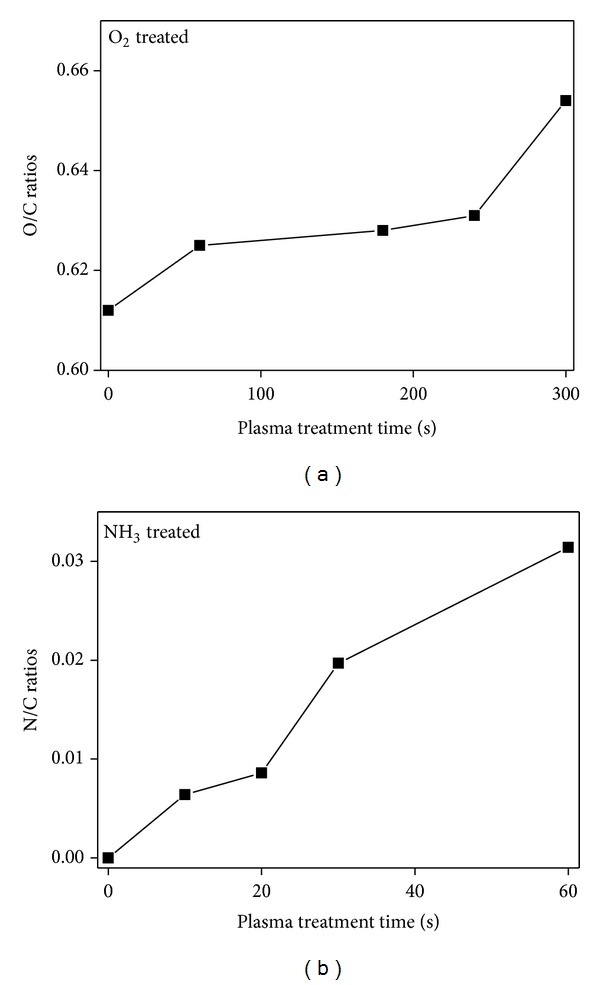
Change in chemical compositions of PLA fiber surfaces with plasma treatment time.

The hybrid electrospun PLA/PLA (20/80) composite fibers were treated with plasma in the presence of either oxygen or ammonia gas to modify the surface properties of the fibers and to further compare the effects of two gases (oxygen and ammonia) on the hydrophilicity of fibers. The morphological changes in the plasma-treated PLA fibers were observed by SEM. No significant changes in the morphology of PLA nano- and microfibers were observed. The change in chemical compositions of PLA fiber surfaces before and after plasma treatment was investigated by XPS (Figure 9). The O/C ratio was increased gradually after oxygen plasma treatment for up to 300 sec. This can be attributed to the formation of hydroxyl or peroxyl groups on the surface of PLA fibers after oxygen plasma treatment. On the other hand, a new N_1s_ peak was observed after treatment with ammonia plasma, indicating newly formed N-containing functional groups such as amines (not shown). Interestingly, the surface of the PLA fibers contained an abundance of nitrogen atoms after ammonia plasma treatment, with an N/C ratio of up to 0.03 (Figure 9).

**Figure 10:**
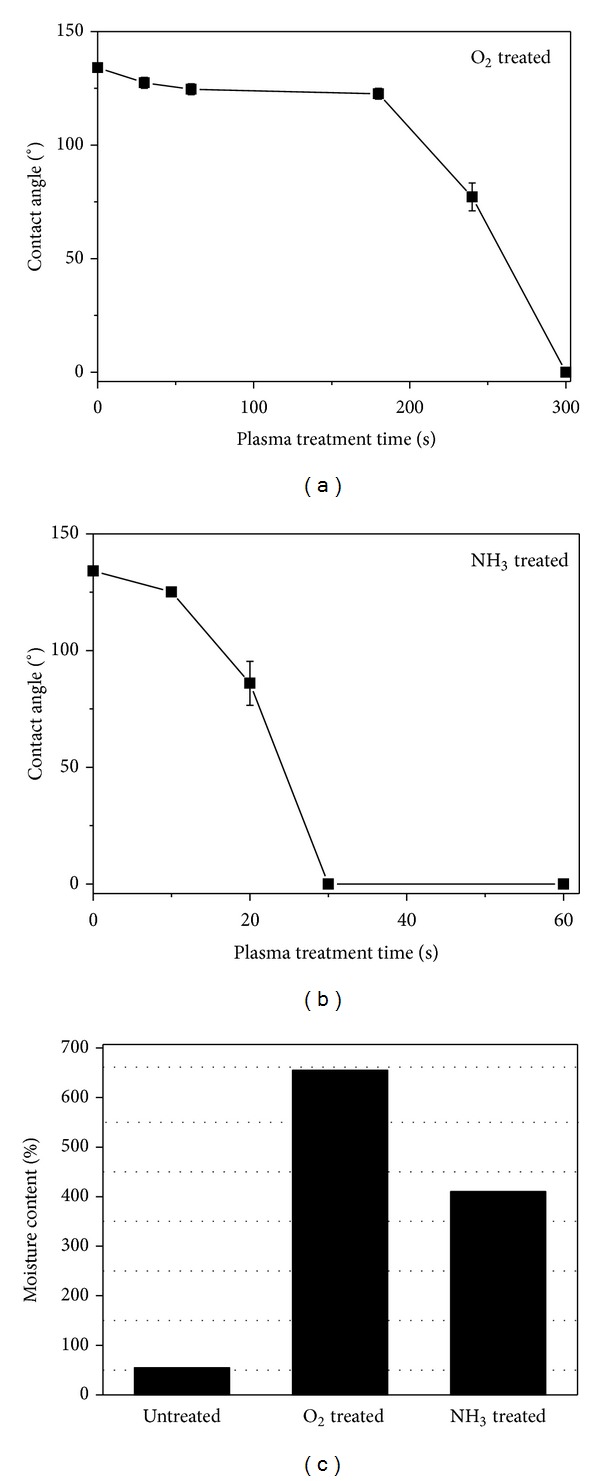
Change in (a) water contact angle (WCA) and (b) moisture content (MC) of PLA/PLA composite fibers with plasma treatment time.

The MC and WCA of plasma-treated PLA/PLA (20/80) composite fibers were also measured to determine changes in the surface hydrophilicity during plasma treatment.  Figure 10(a) shows the change in the WCA of PLA/PLA composite fibers with plasma treatment time. The WCA on the nontreated PLA/PLA composite fibers was 135°, indicating that the surface of the nontreated PLA fibers was quite hydrophobic. This value decreased abruptly after oxygen plasma treatment for 240 sec and reached 0° after 300 sec. In the case of ammonia plasma treatment, the WCA value decreased more quickly than the oxygen plasma and reached 0° after 30 sec. The MC of the nontreated PLA fibers was also increased significantly from 55% to 655% and 410% after treatment with oxygen plasma (treatment time = 240 sec) and ammonia plasma (treatment time = 20 sec), respectively. The reduction in WCA and the increase in MC clearly support the increased surface hydrophilicity of the PLA fibers, which might be caused by the introduction of new polar groups on the surface of the PLA fibers. Furthermore, the ammonia gas plasma enhanced the surface hydrophilicity of PLA fibers more effectively than oxygen gas plasma.

## 4. Conclusion

The effects of plasticizer on the average diameter and morphology of melt electrospun PLA fibers have been examined. The average fiber diameter of the PLA microfibers decreased with increasing PEG plasticizer content due to the lower melt viscosity. Novel composite scaffolds were fabricated to combine the beneficial properties of nanofibers and microfibers. PLA/PLA and SF/PLA nano-/microfiber composite scaffolds were obtained by hybrid electrospinning, in which both the PLA and SF solution and PLA melt produced randomly mixed nanofibers and microfibers. The mechanical properties of the PLA microfibers were improved remarkably by introducing a small amount of nanofibers (20 wt%), even though they had similar pore parameters. The surface hydrophilicity and the content of polar groups on the surface of PLA fibers were increased significantly after plasma treatment in the presence of either oxygen or ammonia gas. This approach to controlling the hydrophilicity and diameters of PLA composite fibers can be useful in the design and tailoring of novel scaffolds for tissue engineering.

## Figures and Tables

**Table 1:** Melt- and hybrid electrospinning conditions of PLA microfibers and PLA composite fibers.

Sample	PEG content (wt%)	Melt electrospinning					Solution electrospinning				
		*T*_*s*_ (°C)	*Q* (mL/h)	*D*_*n*_ (mm)	*V* (kV)	TCD (cm)	*T*_*s*_ (°C)	*Q* (mL/h)	*D*_*n*_ (mm)	*V* (kV)	TCD (cm)
PLA microfiber	0~20	215	0.65~5.40	0.495	21	8	—				
PLA/PLA (20/80) Nano-/microfibers	10	215	1.12	0.495	21	8	25	4	0.495	10	5
SF/PLA (20/80) Nano-/microfibers	10	215	1.12	0.495	21	8	25	4	0.495	10	5

*T*_*s*_: spinning temperature.

*Q*: mass flow rate.

*D*_*n*_: needle diameter.

*V*: applied voltage.

TCD: tip to collector distance.

**Table 2:** Pore parameters and mechanical properties of the PLA microfibers and nano-/microfiber (20/80) composite scaffolds.

Sample	Pore properties			Mechanical properties		
	TIV (mL/g)	APD (*µ*m)	*P *(%)	Tensile strength (gf/mm^2^)	Elongation break (%)	Modulus (gf/mm^2^)
PLA microfiber	13.3	44.7	94.2	1.5 ± 0.2	46.1 ± 6.5	0.3 ± 0.04
PLA/PLA (20/80) Nano-/microfibers	9.9	34.5	91.6	26.8 ± 13.6	20.6 ± 2.9	2.7 ± 0.4
SF/PLA (20/80) Nano-/microfibers	6.4	39.2	95.0	20.6 ± 3.6	11.0 ± 1.5	2.1 ± 0.4

TIV: total intrusion volume.

APD: average pore diameter.

*P*: porosity.
